# Antiviral Activity of TMC353121, a Respiratory Syncytial Virus (RSV) Fusion Inhibitor, in a Non-Human Primate Model

**DOI:** 10.1371/journal.pone.0126959

**Published:** 2015-05-26

**Authors:** Gabriela Ispas, Anil Koul, Johan Verbeeck, Jennifer Sheehan, Brigitte Sanders-Beer, Dirk Roymans, Koen Andries, Marie-Claude Rouan, Sandra De Jonghe, Jean-François Bonfanti, Marc Vanstockem, Kenneth Simmen, Rene Verloes

**Affiliations:** 1 Janssen Infectious Diseases, Beerse, Belgium; 2 Janssen Research & Development, London, United Kingdom; 3 Huntingdon Life Sciences, 100 Mettlers Road, Somerset, NJ 08873, United States of America; 4 BIOQUAL, Inc., 9600 Medical Center Drive, Rockville, MD 20850, United States of America; 5 Janssen Research & Development, Beerse, Belgium; 6 Jan-Cil France, Janssen Research & Development, Paris, French Republic; 7 Jan-Cil UK, J&J Pharmaceutical Research & Development, High Wycombe, United Kingdom; University of Georgia, UNITED STATES

## Abstract

**Background:**

The study assessed the antiviral activity of TMC353121, a respiratory syncytial virus (RSV) fusion inhibitor, in a preclinical non-human primate challenge model with a viral shedding pattern similar to that seen in humans, following continuous infusion (CI).

**Methods:**

African green monkeys were administered TMC353121 through CI, in 2 studies. Study 1 evaluated the prophylactic and therapeutic efficacy of TMC353121 at a target plasma level of 50 ng/mL (n=15; Group 1: prophylactic arm [Px50], 0.033 mg/mL TMC353121, flow rate 2.5 mL/kg/h from 24 hours pre-infection to 10 days; Group 2: therapeutic arm [Tx50], 0.033mg/mL TMC353121 from 24 hours postinfection to 8 days; Group 3: control [Vh1] vehicle, 24 hours post-infection to 8 days). Study 2 evaluated the prophylactic efficacy of TMC353121 at target plasma levels of 5 and 500 ng/mL (n=12; Group 1: prophylactic 5 arm [Px5], 0.0033 mg/mL TMC353121, flow rate 2.5 mL/kg/h from 72 hours pre-infection to 14 days; Group 2: prophylactic 500 arm [Px500], 0.33 mg/mL TMC353121; Group 3:control [Vh2] vehicle, 14 days). Bronchoalveolar lavage fluid and plasma were collected every 2 days from day 1 postinfection for pharmacokinetics and safety analysis.

**Findings:**

TMC353121 showed a dose-dependent antiviral activity, varying from 1log_10_ reduction of peak viral load to complete inhibition of the RSV replication. Complete inhibition of RSV shedding was observed for a relatively low plasma exposure (0.39 μg/mL) and was associated with a dose-dependent reduction in INFγ, IL6 and MIP1α. TMC353121 administered as CI for 16 days was generally well-tolerated.

**Conclusion:**

TMC353121 exerted dose-dependent antiviral effect ranging from full inhibition to absence of antiviral activity, in a preclinical model highly permissive for RSV replication. No new safety findings emerged from the study.

## Introduction

Respiratory syncytial virus (RSV) infection is the most important cause of upper and lower respiratory tract disease in infancy and early childhood. Globally, RSV infections are estimated to result in up to 199,000 deaths annually in children younger than 5 years of age [1]. Prematurely born infants, in conjunction with chronic lung disease, congenital heart disease, and patients with T-cell immunodeficiency have higher risk of developing severe RSV infection [2]. Although RSV is traditionally regarded as a pediatric pathogen, it also causes life-threatening pulmonary disease in immunocompromised adults [3] and in elderly population [4]. Severe RSV pneumonia occur in elderly with co-morbidities or compromised immune status, leading to prolonged hospitalizations and a substantial economic burden on health services [5]. The potential burden of undiagnosed RSV disease in adults on the healthcare system has not been clearly estimated, and is possibly unappreciated [6].

Attempts to develop an effective vaccine to prevent the spread of RSV have been unsuccessful till date. In 2006, American Academy of Paediatric’s guidelines on management of RSV bronchiolitis recommended the use of ribavirin (the only approved RSV antiviral for therapeutic indication) in high risk patients. Ribavirin has a high degree of toxicity, is teratogenic and requires inhalation as preferred mode of administration for respiratory disease. Moreover, ribavirin has low anti-RSV activity (EC_50_ on Hep2 cells, for RSV-A virus: 30 μM) and reduced selectivity index [7], which could explain the controversial effectiveness observed in clinical settings with this drug. Another drug, palivizumab is indicated for prophylaxis in high-risk children, reducing the risk of RSV related hospitalizations. However due to the high costs and the request for multiple intramuscular administrations, the use of palivizumab is restricted to high risk children [8]. Other drugs such as bronchodilators, steroids, antibiotics are commonly used for the treatment of RSV bronchiolitis despite clear studies showing no clinical benefit [9] and they are not indicated for the treatment of RSV bronchiolitis. In general vaccines against RSV have not proved to be safe and effective yet.

The fatal cases associated with a formalin-inactivated RSV vaccine in children [10] and the limited effectiveness of influenza vaccine in the elderly [11], suggest that developing an RSV vaccine in these patients, with immature and respectively deficient immune responses is a major challenge. There is thus an unmet need to develop more efficacious and safe therapeutic options in children and elderly in particular, against RSV infection for both prophylactic and therapeutic indications.

TMC353121, a small substituted benzimidazole RSV fusion inhibitor, has been developed by molecular modeling of the precursor molecule JNJ-2408068. It maintains high activity (pEC50 9.9) and low cytotoxicity, while presenting a shorter half-life in the lung (lung t_1/2_ 25 h) [12]. TMC353121 binds to an intermediate conformation of F. Binding of TMC353121 stabilizes the interaction of HR1 and HR2 in an alternate conformation of the 6HB, in which direct binding interactions are formed between TMC353121 and both HR1 and HR2. Rather than completely preventing 6HB formation, data indicate that TMC353121inhibits fusion by causing a local disturbance of the natural 6HB conformation [13].

The compound showed significant antiviral activity in rodent models of RSV infection [14] when administered as a slow single bolus injection [14], leading to a suboptimal but efficacious drug exposure.

The current study was undertaken to assess the efficacy of TMC353121 in conditions of optimum drug exposure, when administered under continuous intravenous infusion (CI), in a non-human primate model with RSV replication characteristics similar to that seen in humans.

## Methods

### Animals

The study was done at BIOQUAL, an USDA-licensed research facility, accredited by the Association for Assessment and Accreditation for Laboratory Animal Care International. The animals were selected from a stock of African Green Monkeys (AGM) purchased from Three Springs Scientific, Inc., Perkasie, PA. African green monkeys were chosen because they are the most commonly used nonhuman primate model for RSV infection. The animals were wild-caught and were imported from St. Kitts Island by Three Springs Scientific, Inc. Upon arrival they were placed on six-week quarantine according to federal regulations. Adult animal were used (exact age was not determined, because the animals were wild caught), with a weight of 5.9 ±0.2 kg. During the quarantine, the animals received an entrance physical examination, which included Complete Blood Count (CBC), blood chemistry profile, serology, fecal flotation, a tuberculin test, and other examinations deemed necessary by the attending veterinarian. All animals were single-housed according to AAALAC and United States Department of Agriculture (USDA) standards, in a modular caging system (Primate Products Inc.). Each module contains two 9.0 cubic feet compartments, one above the other. The BIOQUAL Environmental Enrichment Program for nonhuman primate housing provides an adequate living space, visual, auditory and olfactory contact with conspecifics, food variety. Treat items offered at multiple feeding times each day, climbing devices, manipulanda, and foraging devices are all utilized to provide an enhanced environment and to promote psychological wellbeing. Feed was supplied by Harlan, peanuts were supplied by Barcelona Nut Shop and fruit and vegetables by L&M Produce. Teklad 5038 Primate Diet was provided once daily by animal size and weight and the diet was supplemented with fresh fruit and vegetables. Fresh water in water bottles was given at least three times daily.

The animals were kept in separate rooms upon infection in standard stain-less-steel monkey cages. For all procedures, the animals were anesthetized intramuscularly with 10 mg/kg ketamine. Each day, the animals were observed, their food consumption was monitored, and their body temperatures were measured.

Animals were screened for the presence of RSV-specific antibodies using serum neutralization antibody assay and were placed on six-week quarantine. The RSV status was assessed again, after the end of the quarantine and immediately prior to study initiation. Housing and handling of the animals were in accordance with the standards of the Association for Assessment and Accreditation of Laboratory Animal Care, the Animal Welfare Act as amended, and the Public Health Services Policy. Handling of RSV-infected samples and animals occurred in compliance with the Biosafety in Microbiological and Biomedical Laboratories. The local ethical committee in compliance with the Declaration of Helsinki approved all studies.

### Study drug

The drug was administered as continuous infusion, through a central catheter. The monkeys were preanesthetized with ketamine and anesthetized via isoflyrane anesthesia for insertion of the central catheter. Each animal was shaven in the inguinal and dorsal regions. An antiseptic was applied to the shaved regions, and the animal was draped for the surgical procedure. A small incision was made in the right inguinal region, and the femoral vein was isolated. A sterile medical-grade polyurethane catheter (5 French, 7 foot, polyurethane catheter with rounded tip; catalog #CNC- 5P-RT-7 s (Access Technologies, Skokie, IL 60076) was inserted into the vessel and advanced into the inferior vena cava region, just caudal to the kidneys. The base of the catheter was secured with four sutures around the femoral vein and a sutured subcutaneous loop. The remainder passed subcutaneously to exit on the right flank of the animal. Both regions were closed by sutures. Since the catheter was not radio-opaque, no radiography was performed after insertion. The exterior part of the catheter was attached via an injection port to a Baxter infusion line (103” IV sets for Baxter infusion pumps) which was connected to a 500 mL infusion bag that was operated by either a BAXTER 6300 or BAXTER 6301 dual channel pump. Analgesics were administered preemptively (Ketofen 5 mg/kg) in addition to atropine (0.02 mg/kg). Antibiotics (Baytril 5 mg/kg) were administered for five days following surgery. The central catheter (medical-grade polyurethane catheter) was inserted into the femoral vein and advanced into the inferior vena cava. Immediately following surgery, sterile saline was infused at a rate of 10.0 mL/hour prior to switching to active drug or placebo. Saline infusion was continued until initiation of dosing.

The test substance, TMC353121 was provided in 10 mL glass vials in 12% Captisol at pH 6.0 at a concentration of 10 mg/mL. To generate infusion bags (500 mL containing 4% Captisol with concentrations of 0.3mg/mL or 0.033 mg/mL or 0.0033 mg/mL of TMC353121, a volume of 0.165mL or 1.65 mL or 16.5 mL of the 10 mg/mL stock solution was injected into the infusion bag. The bags were then inverted 20 times for mixing. The control group was infused with only vehicle, consisting of 4% Captisol at pH 6.0, isotonic with saline.

### Study design

A block design was applied with one animal of each group in a block and a randomization table provided by statistician was used to allocate the animals into their respective blocks and treatment groups. The animals selected for the studies were sorted by weight, with animals within a block having similar weights. Once the animals had been selected for each block, they were arranged by their weights in ascending order.

#### Study 1

Study 1 (Table 1) evaluated the prophylactic and therapeutic efficacy of a CI of TMC353121 at a target plasma level of 50 ng/mL. Total of 15 animals were divided into 3 groups- Group 1: the prophylactic arm (Px50), received a 0.033 mg TMC353121/mL solution at a flow rate of 2.5 mL/kg/h, starting 24 hours before infection daily, for a total duration of 10 days; Group 2: the therapeutic arm (Tx50), received the same dose, starting 24 hours postinfection, for a total duration of 8 days; Group 3: the control arm (Vh1), received the vehicle (4% aqueous Captisol), starting 24 hours after infection, for a total duration of 8 days. In Study 1 all animals were followed until day 13 post-inoculation, when the necropsy took place.

**Table 1:** Flow chart Studies 1 and 2.

Day			-3 to-1	0	1	2	3	4	5	6	7	8	9	10	11	12	13	15
RSV Infection				X														
**Continuous Infusion**	**Study 1**	**Vh1 (n = 5)**			X	X	X	X	X	X	X	X						
	**Study 1**	**Px 50 (n = 5)**	X	X	X	X	X	X	X	X	X	X						
	**Study 1**	**Tx 50 (n = 5)**			X	X	X	X	X	X	X	X						
	**Study 2**	**Vh2 (n = 4)**	X	X	X	X	X	X	X	X	X	X	X	X	X	X		
	**Study 2**	**Px 5 (n = 4)**	X	X	X	X	X	X	X	X	X	X	X	X	X	X		
	**Study 2**	**Px 500 (n = 4)**	X	X	X	X	X	X	X	X	X	X	X	X	X	X		
**Sample collection: BAL,** ^1^ **swabs, plasma for drug level**					X		X		X		X		X		X		(X)	
**Necropsy**																	X	X

Groups for Study 1: Vh1: vehicle; Px 50: prophylactic treatment with TMC353121 50ng/mL targeted plasma concentration; Tx50: therapeutic treatment with TMC353121 50ng/mL targeted plasma concentration. Groups Study 2: Vh2: vehicle; Px5: prophylactic treatment with TMC353121 5ng/mL targeted plasma concentration; Px500: prophylactic treatment with TMC353121 500ng/mL targeted plasma concentration. Colored boxes indicate duration of the event corresponding to the color. X indicates one of the following: an event took place on specified day (e.g.: RSV infection, necropsy) or specified biological specimens (BAL, swabs) were collected on specified days. (X): BAL and swab sampling on day 13 were done just for Study2. Sampling Jacket and tether training and catheter surgery are not included in the flow chart. Vh1 and Vh2: 4% aqueous Captisol; BAL: bronchoalveolar lavage.

^**1**^Baseline BAL analysis was performed 3 d prior to RSV challenge. Sample collection was performed every second day, starting at day 1 post- challenge. Necropsy was performed on Day 13 for Study 1 and on Day 15 for Study 2.

#### Study 2

Study 2 (Table 1) evaluated the prophylactic efficacy of a CI of TMC353121 at target plasma levels of 5 and 500 ng/mL. Total of 12 animals were divided into 3 groups- Group 1: the Prophylactic 5 arm (Px5), received a solution of 0.0033 mg/mL TMC353121, at a flow rate of 2.5 mL/kg/h, starting 72 hours before infection, for a total duration of 16 days; Group 2: the Prophylactic 500 arm (Px500) received 0.33 mg/mL TMC353121; Group 3: the control arm (Vh2) received vehicle (4% aqueous Captisol) during the same time span (16 days). In Study 2 all animals were followed until day 15 post-inoculation, when the necropsy took place.

### Sample Collection

Bronchoalveolar lavage fluid (BALF), nasal and throat swabs and blood samples were collected every 2 days from day 1 postinfection up to day 11 (study 1) and day 13 (study 2). For BALF collection, monkeys were sedated with atropine (0.02–0.05 mg/kg) administered intramuscularly to allow passage of sterile laryngoscope and endotracheal tube. BAL was performed by laryngoscopy by inserting a shortened Kendall Sovereign feeding tube and urethral catheter through the endotracheal tube and approximately 10 mL of phosphate buffer saline (10 mL, pH 7.4) was instilled and gently suctioned back into the syringe promptly to recover approximately 5–7 mL of BAL in the syringe. Later, the adjustment of the dilution was done based on urea and protein concentration. White blood cell (WBC) count from BALF was analyzed using automated blood analysis instrumentation (Antech Diagnostics, Lake Success, NY, USA).

For nasal and throat swabs collection, the swab tip was pre-wetted in a solution of 1.2 mL Eagle's Minimal Essential Medium (EMEM), 5% Fetal Calf Serum (FCS), Amphotericin B (2.5 μg/mL) and Penicillin-Streptomycin (100 IU/mL: 100 μg/mL). While collection, the tip was squeezed against the tub in which the tip had been dipped, returning as much of the liquid to the original tube as possible. The tip was then clipped, so that it would fit into a 2 mL cryotube. Peripheral blood sample was collected by venipuncture of the femoral vein for urea and drug analysis. All the samples collected were aliquoted and stored at -80°C until analysis except for WBC determinations.

### RSV Serology: Serum Neutralization Antibodies Assay

Monkeys were tested for the presence of RSV neutralization antibodies in serum at enrollment, after the end of the six-week quarantine and immediately prior to the study start. Serial 2-fold dilutions of heat-inactivated serum were assayed for the presence of neutralizing antibody in a microneutralization assay starting with a dilution of 1:10 (1:10, 1:20, 1:40, 1:80, 1:160, etc.), incubated at 37°C for 1 hour with 1mL diluted virus (100 PFU/mL). Virus-serum mixtures were transferred to 1 day old Vero cell monolayers in 96-well plates and incubated at 37°C for 7 days, after which all wells were observed for cytopathic effect (CPE). The serum dilution that completely neutralized infectivity of RSV was calculated by previously described method [15,16].

### Cell culture and RSV Preparation

RSV A2 virus and the Vero cells were obtained from ATCC (Rockville, MD, USA). Vero cells were propagated in duplicate wells containing 1 day old monolayer cell culture (plating ratio was one confluent T-75 flask to 150 mL). The virus propagated in Vero cells and infectious RSV titers were determined by plaque assay as previously described [17] and by quantitative reverse-transcriptase polymerase chain reaction (qRT-PCR) assay as described below.

### RSV Challenge

All animals were challenged with 1.5mL of 1 × 10^4^ pfu/mL RSV subgroup A—strain A2 virus intranasally (i.n.) and intratracheally (i.t.) on day 0. The infectivity and replication kinetics of RSV A2 challenge stock virus were confirmed by prior titration study in AGMs at the Tulane National Primate Research Center. The titer of 104 PFU/mL and the inoculation method was determined in a viral titration study as the minimal dose and optimum condition to ensure that the AGMs would be productively infected. The virus was thawed at room temperature and immediately put on ice.

For each inoculation, 1.5 mL were aspirated with a 3 mL syringe. After being anesthetized with 10 mg/mL ketamine, the animals were placed in dorsal recumbency. 0.25 mL of the virus was instilled drop-by-drop into each nostril and allowed to absorb for 20 sec. Once the absorption was complete, a Kendall Sovereign Feeding Tube and Urethral Catheter, Size 5 Fr, was trimmed about one inch on each side and inserted intratracheally using a laryngoscope. Once in place, 1mL of virus was instilled and flushed into the trachea with 7 mL of air.

### Viral Load Determination

RNA was extracted using a previously described protocol [18]. The External Quantification Control (EQC) standards were constructed using RNA transcripts of pEQC-A (Cloned fragment from RSV-AGFP (GST011828). RSV was quantified in biological samples by detection of RSV-A F gene. The primers used for RSV-A F gene were: RSVA-F-FW656: 5’-CTGTGATAGARTTCCAACAAAAGAACA-3’ and RSVA-F-RV732: 5’-AGTTACACCTGCATTAACACTAAATTCC-and RSVA-F-TP684: FAM 5’- CAGACTACTAGAGATTACC-3' NFQ-MGB. For internal extraction control the RSV-B N gene was used (pIEC-B). The primers used for RSV-B N gene were: RSVB-N-FW435: 5’-GGCTCCAGAA-TATAGGCATG-ATTC-3’; RSVB-N-RV508: 5’-TGGTTATTAC-AAGAGCAGCT-ATACACAGT-3’ and RSV-B-EC-Ngen-TP: FAM 5'-TACCGTACTCTAGCCTA-3' NFQ-MGB.

RSV was quantified using one-Step RT-PCR Master Mix reagents (Applied Bio systems, USA). Amplification and detection were performed in a ABI PRISM 7700 Sequence Detection System (Applied Biosystems, USA) under the following conditions: an initial reverse transcription at 48°C for 30 minutes, followed by PCR activation at 95°C for 10minutes and 45 cycles of amplification (15 sec at 95°C and 1minutes at 60°C).

The IEC-B assay was run as control. The viral load (VL) was expressed as RSV A-F copy numbers and transformed in Δ log10 values. The limit of detection was 10 copies of RSV F gene. The antiviral effect was calculated as differences between peak VL and area under the curve (AUC) VL based on Δ log10 RSV from different groups.

### Cytokine Determination

The following monkey-specific cytokines were determined in BALF using a Luminex bead analysis according to the manufacturer's protocol: Interleukins (IL)-1b, IL-2, IL-4, IL-5, IL-6, IL-8, IL-10, Interferons (IFN)γ, IFNα, Tumor necrosis factor (TNF)α, Regulated on Activation, Normal T Cell Expressed and Secreted (RANTES), and Macrophage inflammatory protein (MIP)-1α. The non-human primate Luminex beads for IFNγ, IFNα, and IL-10 were provided by Texas Biomedical Research Institute (Texas, USA). The beads for the remaining cytokines were obtained from Millipore (Billerica, MA, USA). The samples for cytokine determination were run in duplicates. Human cytokine standards were used for standard curves and for determination of the lower limit of detection for individual cytokines. The results were read using a Luminex 100 System (Texas, USA).

### Bioanalysis of TMC353121

Blood samples for PK assessment were collected every second day, starting with day 1 post viral inoculation. Plasma samples were analyzed individually for TMC353121 by high performance liquid chromatography (HPLC) (Agilent, CA, USA) coupled to quadruple mass spectrometry detection (Qualified Research Liquid Chromatography Tandem Mass Spectrometry [LC-MS/MS] method) (API-3000 MS/MS [Applied Biosystems, CA, USA]). The limit of detection for TMC353121 was 0.19 ng/mL. The concentration of drug in different compartments was expressed as fold EC_50_ applied to the measured drug concentration in plasma, lung and undiluted BAL.

### Safety Analysis

The animals were monitored for clinical symptoms such as body temperature changes indicating a fever, signs of a cold, runny nose, sneezing, loss of appetite, and body weight. Blood counts and serum chemistry were assessed every second day. The safety findings were compared between the vehicle groups and the compound groups, to identify possible compound related adverse events.

### Histopathology

Tissues (administration site, heart, liver, lung, pancreas, skeletal muscle and trachea) were fixed in 10% phosphate-buffered formalin, paraffin-embedded, sectioned at 5 μm, stained with hematoxylin-eosin (H&E) and evaluated microscopically. For the lung, histological examination was performed on the right lung only, the left lung lobe was sampled for bioanalytical purposes. At tissue trimming, representative cross sections were taken from the cranial, mid and caudal lung lobe in all animals including the central part (with large bronchus) and the peripheral part. Three lung sections per animal (cranial, mid and caudal lobe) with two samples each (central and peripheral) were evaluated for each animal. To facilitate comparison between groups, a general overall score for inflammation was given for each lung section. Mean inflammatory scores were determined for each animal (mean of 3 lung lobes) and per dose group (S2 Fig).

### Necropsy

The necropsy was performed at day 13 for Study 1 and at Day 15 for Study 2. Before necropsy, a sample for complete blood count and plasma sample was taken. Euthanasia procedures adhered to the current Report of the American Veterinary Medical Association Panel on Euthanasia. The anesthesia was performed with a combination of ketamine hydrochloride (7–15 mg/kg) and either acetylpromazine maleate (acepromazine) (0.55 mg/kg) or xylanin (Rompun) (0.6 mg/kg) followed by exsanguinations via the femoral vein. The blood taken was recorded. Thereafter, an injection of 390 mg/kg sodium pentobarbital intravenously was given. Death was defined as the cessation of respiration and of heartbeat. The necropsy was performed according to BIOQUAL SOPs and a board-certified veterinary pathologist from the Charles River Laboratories, Pathology Associates Division (PAI) was present at necropsy.

### Statistical Analysis

Mean (±SEM) or median (minimum, maximum and coefficient of variation) were calculated for viral kinetics parameters (peak and AUC VL), drug exposure and cytokines. Differences between groups were assessed with Kruskal Wallis test for nonparametric comparison of three or more unpaired groups and Dunn`s posttest for comparison between each pair of groups, using the vehicle treated group as reference for individual studies. A p< 0.05 was considered significant. Correlation coefficients and p values for correlation analysis were calculated using Spearman nonparametric two-tailed, linear regression. Analysis was performed with GraphPad Prism, version 4 (GraphPad Software, San Diego, CA).

## Results

### Pharmacokinetics

**Fig 1:**
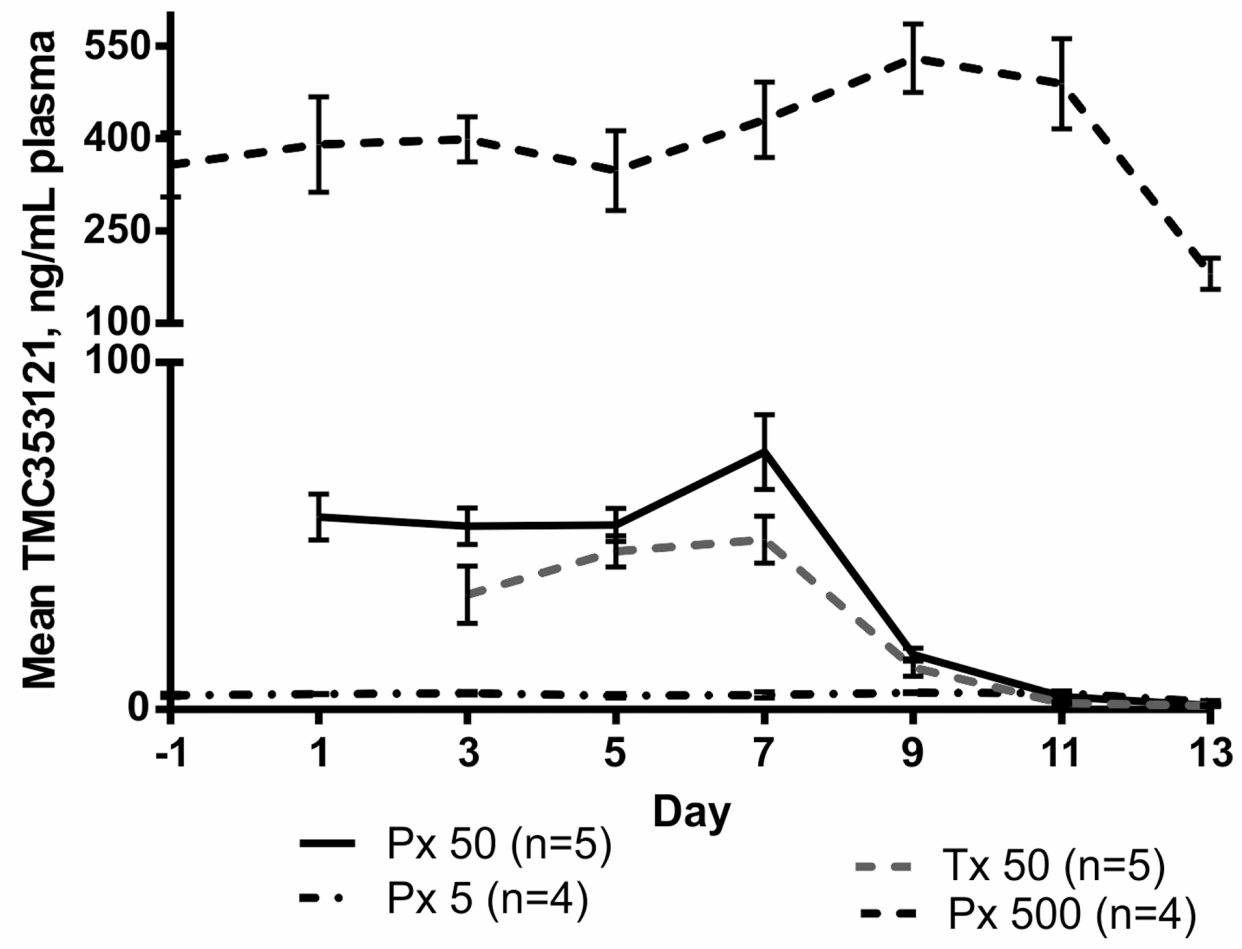
Pharmacokinetics of TMC353121 administered under continuous infusion. Mean ± SEM TMC353121 plasma concentrations are presented for Studies 1 and 2.

One AGM in the control group (V1) was accidentally infused with the drug for 24 hours, two days after infection (Table 2). Conversely, one monkey in the Tx50 group had a 24 h drug holiday two days after infection. The steady state mean plasma level after 1 day of infusion was of 32 ng/mL for the Px50 group, 20 ng/mL for the Tx50 group, 4.12 ng/mL for the Px5 arm and 390 ng/mL for the Px500 arm (Fig 1, Table 2). After cessation of the infusion, the plasma concentration decreased steadily, but was still detectable at the time of necropsy in the highest dose group. The drug distribution in BALF and plasma compartment showed a linear kinetics, with dose- dependent increase in exposure from Px5 to Px500 arm (Fig 1, Table 2). However, the distribution of TMC353121 in lung tissue was dose linear only between Px50 and Px500 arms and not between Px5 and Px50 arms. Across the two studies, the drug was present in a range of 0.5 to 68 folds EC_50_c in BALF and plasma, and in a range of 8 to 3300 folds EC_50_c in the lung compartment (Table 2).

**Table 2:** Tabular results of TMC353121 pharmacokinetics.

Matrix	TMC353121	Study 1	Study 1	Study 1	Study 2	Study 2	Study 2
		Vh1 (n = 5)	Px 50 (n = 5)	Tx 50 (n = 5)	Vh2 (n = 4)	Px 5 (n = 4)	Px 500 (n = 4)
**Plasma**	Mean± SEM(ng/mL)	7.4±5.9	32.1±10.5	20.4± 8.1	<LOD	4.1± 0.3	390.6±373
	Fold EC50c	1	4.5	2.8	<LOD	0.5	55.8
**BALF**	Mean± SEM(ng/mL)Urea corrected	6.1±2.2	32±7.7	24.2±6.7	<LOD	4.7±0.7	331±67.9
	Fold EC50c	0.8	4.5	3.4	<LOD	0.6	47.2
**Lung**	Mean± SEM(ng/g)	<LOD	231.8± 51	59± 13.1	<LOD	174±47.7	23267±7159
	Fold EC50c	<LOD	33	8.4	<LOD	24.8	3323

The concentration of drug in different compartments was expressed as fold EC50c. EC_50c_ = 7 ng/mL x plasma protein binding correction factor: 100. Urea dilution factor of 9.7 was applied for drug concentration in BALF. LOD = limit of detection. LOD_TMC353121_ = 0.19 ng/mL.

### RSV Serology

At all study time points, the results were negative in study 1 and 2, indicating that the participating animals had no prior RSV exposure.

### Viral Shedding

**Fig 2:**
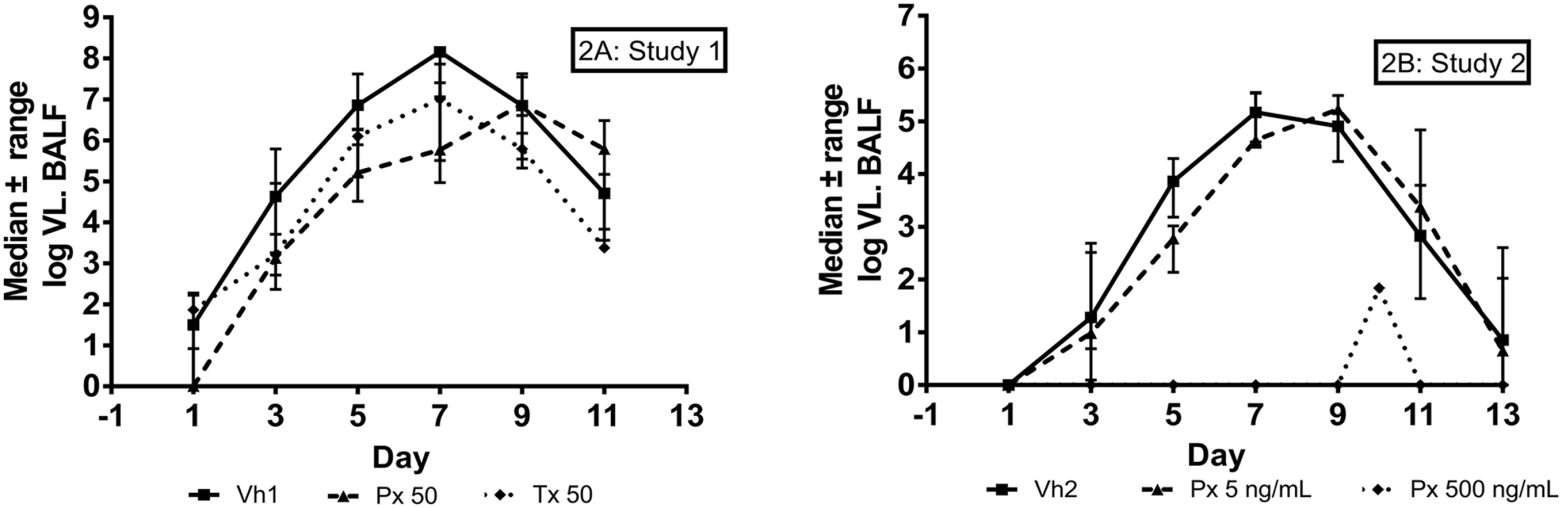
Viral kinetics in BALF. Viral load assessed by qRT-PCR was expressed in RNA copies per ml. The time course of viral load (Median± IQR) is shown for Study 1 and Study 2.

In study 1, the Vh1 arm had the highest peak VL (8.15 log_10_), followed by the Tx50 (7 log_10_) and Px50 arm (6.88 log_10_) (Fig 2A, Table 3). The therapeutic and prophylactic treatment with TMC353121 resulted in a similar decrease in peak VL in BALF compared with the control group (1.27 log_10_ for Px50 and 1.15 log_10_ for Tx50). Moreover, the reduction of VL AUC was higher for the Px50 arm (20%) compared with the Tx50 arm (5%) (Table 3). In addition, 3 out of 5 animals in the prophylactic arm had a delayed peak viral shedding from day 7 to day 9 (Fig 2A). Overall, the time course of the viral shedding was the same among all 3 groups, with the VL dropping below the lower limit of quantification by day 15 postinfection.

**Table 3:** Antiviral activity in BALF based on reduction of peak VL (qRT-PCR data).

Viral kinetics	Study 1	Study 1	Study 1	Study 2	Study 2	Study 2
	Vh1 (n = 5)	Px 50 (n = 5)	Tx 50 (n = 5)	Vh2 (n = 4)	Px 5 (n = 4)	Px 500 (n = 4)
**Peak log VL (Median)**	8.15	6.88	7.00	5.17	5.23	0
**CV (%)**	8.79	17.48	11.79	5.06	8.72	200.00
**Δ log (Median)**	NA	-1.06	-1.1	NA	0	-5.26
**P**	>0.05	>0.05	>0.05	<0.05	>0.05	<0.05

BALF: bronchoalveolar lavage fluid; VL: viral load; CV: coefficient of variation. The comparison between groups from individual studies is expressed as Δ log_10_ and peak VL (median); p values were computed based on Kruskal Wallis test; Δ log_10_ VL: reduction in peak VL (median) for drug-treated group compared with vehicle treated group.

In study 2, animals in the Px500 arm showed complete inhibition of viral replication, during the entire study, except one animal who showed a viral blip at day 10 postinfection (Fig 2B). This animal had the drug infusion stopped at day 9, one day prior to euthanasia (taking place at day 10). VL in BAL was below the limit of detection at day 9 in presence of the drug and was positive (2log) at day 10 (the day of euthanasia), 24h after the drug infusion was stopped.

The viral shedding in throat samples correlated significant (r = 0.79) with the viral shedding in the BALF compartment (S1 Fig). The antiviral effects seen in presence of different TMC353121 drug regimens were similar in throat and BALF compartment.

### Cytokines

**Fig 3:**
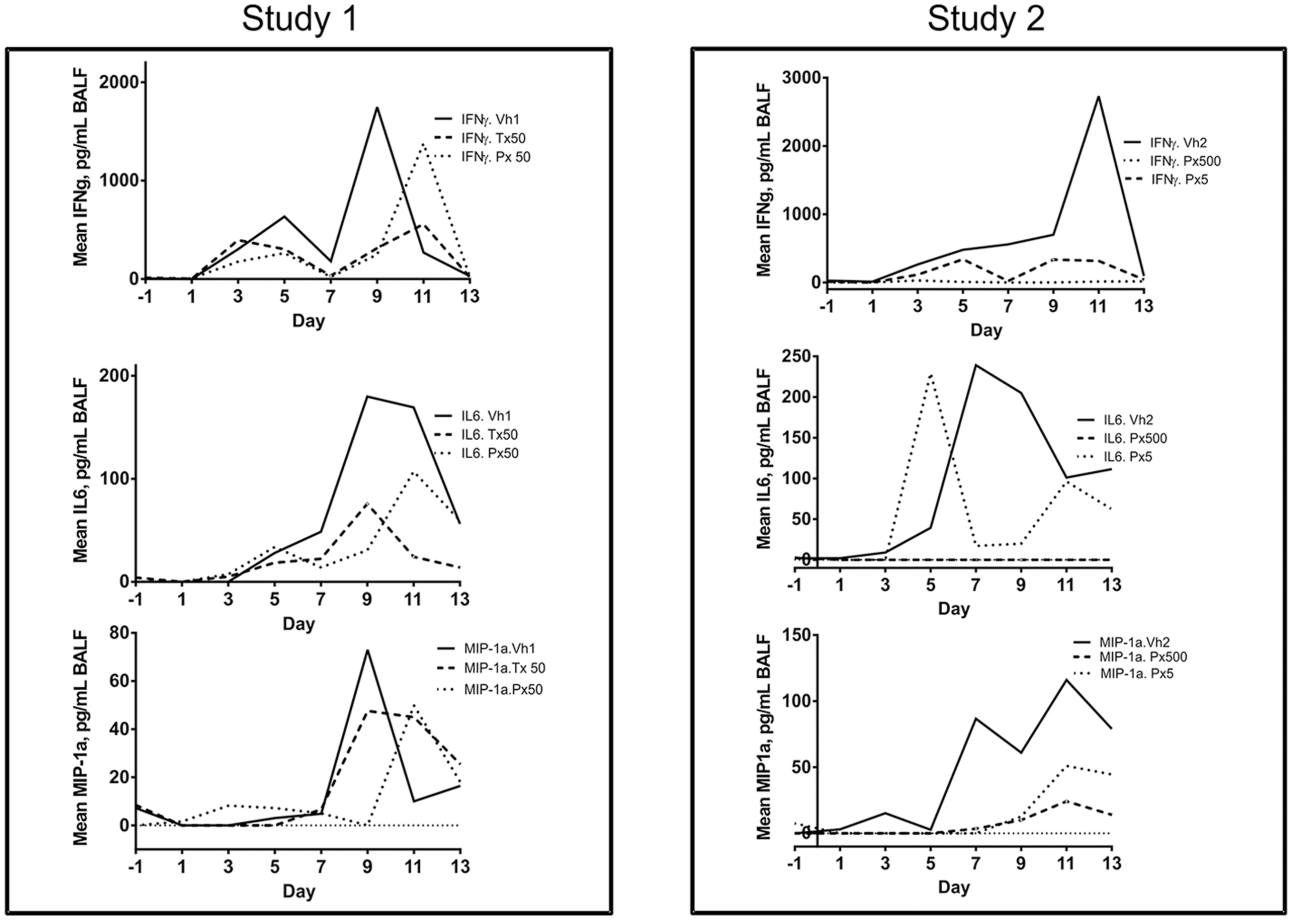
Time course of individual cytokine in BALF. Mean values are shown for IFNγ, IL6 and MIP1a.

The cytokines dynamics was similar in all groups in both studies, with peak values observed between day 9 and 13 postinfection (Fig 3). Though high inter-participant variability was observed, the median and mean values indicated a clear pattern for all individual cytokines tested. The highest values were measured for IFNγ, followed by IL-6, IL-8, and MIP-1α (Fig 3). There was no significant activity above the lower limit of quantification for the following cytokines: IL-1b, IL-2, IL-4, IL-5, and IL-10 and no significant reduction in IL8 production between control and treated animals. In general, there was reduced production of IFNγ, IL-6 and MIP-1α in presence of TMC353121, with an AUC reduction ranging from almost complete inhibition observed for the Px500 arm (eg: 0.5% of control AUC for IFNγ) to similar AUC observed for the Px50 arm (Table 4).

**Table 4:** Median AUC for IFNγ, IL6 and MIP1α in BALF-Comparison between groups from individual studies expressed as % reduction in median AUC.

Cytokines	Parameters	Study 1	Study 1	Study 1	Study 2	Study 2	Study 2
		Vh1 (n = 5)	Px 50 (n = 5)	Tx50 (n = 5)	Vh2 (n = 4)	Px 5 (n = 4)	Px 500 (n = 4)
**IFNγ**	AUC Median	5936	5678	2718	8120	1498	46
	CV (%)	55.7	84.7	73.3	71.7	99.3	147.4
	AUC (%)	100	95.65	45.70	100	18.40	0.56
**IL6**	AUC Median	540	527	391	1174	537.5	0
	CV (%)	78.8	49	93.5	60.8	116.1	173.2
	AUC (%)	100	97.59	72.40	100	45.70	0
**MIP1α**	AUC Median	394	201	106	718.5	142	0
	CV (%)	87.2	152.2	81.6	33.6	88.3	173.2
	% AUC	100	51.00	26.90	100	19.76	0

BALF: bronchoalveolar lavage fluid, AUC: area under the curve, CV: coefficient of variation, % AUC: % reduction in median AUC for drug treated group compared with vehicle treated group. p values were computed based on Kruskal Wallis test. All p values were >0.05.

### Clinical Observations and Pathology

Based on clinical symptoms, blood counts and serum chemistry, no drug related adverse events were observed when vehicle treated groups were compared with compound treated groups. Two animals in the Px500 arm discontinued the treatment, one at day 2 of dosing and the second prior to the last day of dosing, due to adverse events (stomach rupture [n = 1] and inguinal edema with peritonitis [n = 1]). These findings were considered not related to TMC353121 based on toxicological reports.

In the animal with severe peritonitis, very pronounced histopathological lesions were seen at the injection site with subsequent peritonitis, but since these severe changes were in contradiction to only minimal to slight changes at the injection site in the other animals of this study and the previous studies, a primary test article related pathogenesis of the lesions at the injection sites was excluded and other factors unrelated to dosing (malformation/weakness of the vessel wall) appeared likely and might explain an increased sensitivity of this animal to vascular irritation.

Overall, neither RSV infection nor treatment with TMC353121 as CI for 16 days had a major clinical effect on the animals that completed the study, indicating a good safety profile for this compound. Some depressed behaviour was observed when the animals were jacketed and tethered; however, such behaviour disappeared after the restraining equipment was removed.

There were no clinical symptoms of reduction in body weight, fever, or nasal discharge and no obvious differences in food or water intake or animal behavior between treated and control animals. Blood counts and serum chemistry did not reveal any abnormal values except of highly elevated creatine phosphokinase and C-reactive protein, probably related to the experimental procedures.

### Lung Histopathology

Histological examination revealed infection-related findings only in the lungs, showing very pronounced (inflammatory) changes in the vehicle control group and Px5 group, varying from slight to marked degree, and much less pronounced changes in the Px500 group, varying from minimal to occasionally slight degree (S2 Fig). No improvement on lung histopathology was observed for Tx50 and Px50 groups. Other examined tissues demonstrated no obvious treatment-related differences.

## Discussion

This paper presents the prophylactic and therapeutic properties of TMC353121, a small molecule RSV fusion inhibitor, under optimal conditions of compound exposure, in a preclinical model highly permissive for RSV replication.

The mechanism of action of TMC353121 has been shown to be associated with the blocking of viral fusion with the target cells [12, 13]. Similar to other fusion inhibitor antivirals, TMC353121 does not destroy the target pathogen (the virus); instead it inhibits the RSV entry into the host cell, reducing the amount of virus shed and having in consequence an antiviral effect.

This is the first report of complete inhibition of viral shedding with an RSV fusion inhibitor (Px500 group), in AGM, an in vivo model highly permissive for RSV replication [19, 20]. Complete inhibition of the viral shedding was achieved for animals in Px500 group, at plasma exposure levels superior to 55-fold EC_c50_, confirming that complete inhibition of RSV replication can be achieved by blocking the virus entry into the host cells. On the other hand, previous published data with RSV fusion inhibitors in rodents [21] and monkeys [19] had showed only a reduction in VL, and not complete inhibition of viral replication.

Notably, complete inhibition of the RSV shedding was observed in animals in the Px500 arm for a relatively low plasma exposure (0.39 μg/mL) of TMC353121, compared with palivizumab studies in which higher exposure levels were required to achieve 50% prophylactic effectiveness in pediatric patients (37–72 μg/mL) or achieve a complete inhibition of the RSV shedding in cotton rat model (40 μg/mL) [22,23]. The clinical relevance of this finding suggests further assessments of TMC353121.

Therapeutic (+1day postchallenge) or prophylactic administration (2-3day prior challenge) at exposure levels 3- to 5-fold above the EC_50c_ led to 1 log_10_ peak VL reduction, whereas a delay of 2 days in peak viral shedding was observed for prophylactic administrations, in presence (Px50) or absence (Px5) of a significant antiviral activity.

One AGM in the control group (V1) was accidentally infused with the drug for 24 hours, two days after infection. This accidental infusion resulted in a 1.5 log_10_ reduction in peak viral load in BALF, compared to the remaining controls. Conversely, one monkey in the Tx50 group had a 24 h drug holiday two days after infection and showed a viral shedding similar to the other animals in the same group (individual data not shown). Thus, the one day accidental drug holiday did not have an impact on viral shedding whereas the 24h accidental drug administration led to a 1.5 log_10_ reduction in BALF peak viral load, compared to the animals from the same group.

Since AGMs did not develop clinical signs upon RSV infection, the impact of the 1 log_10_ decrease peak VL (observed for Tx50 and Px50 arms) on clinical symptoms could not be evaluated. Nevertheless, data published for the RNAi based therapy indicates that 1log_10_ peak RSV VL reduction might translate in a significant reduction of the clinical symptom score in RSV human challenge model [24].

The kinetics of cytokines followed the viral kinetics, with the highest values measured at viral peak concentrations. Therapeutic and prophylactic interventions reduced the production of total INFγ, IL-6 and MIP-1α in BALF and a remarkable inhibition of cytokines production was observed in the Px500 arm, indicating the absence of active lung inflammation in these animals. The reduction of IL-6 and MIP-1α observed in BALF are of specific interest, due to the reported direct correlation of IL-6 and MIP-1α with clinical symptoms in animal and human RSV lung disease [25–27].

As evident from earlier studies, the RSV non-human primate model does not develop upper and lower respiratory tract illness symptoms as observed in humans [23,24]. Therefore in these studies, the evaluation of the antiviral effect of the compound was restricted to determination of VL, cytokines and histopathology.

Similar to the average infectious dose used in human studies, the viral inoculum required to reach 100% infectivity in monkeys was 10^4^pfu/mL RSV [28]. Moreover, the magnitude and the dynamics of the virus shedding in monkeys was similar to human, with the peak VL reached at 7–9 days post RSV challenge [29].

Limited to no improvement on lung histopathology for Tx50 and Px50 groups in presence of a significant antiviral effect (1log delta peak log VL) does not align with previous published data, which reported improved lung histopathology in mice following single administration of TMC353121, in presence of a significant antiviral effect of 0.5–1log in treated compared with untreated groups [14,30]. Possible explanation of these differences may be due to the frequent BAL procedures (every 2 day), which disturbed the local equilibrium between virus, cellular response and antiviral activity, and caused supplementary lung damage, masking the effect of the antiviral treatment on lung histopathology. This hypothesis is further supported by the observation in humans where the BAL procedure is linked with lung inflammation [31].

TMC353121 administered as CI for 16 days was well-tolerated, i.e. no clinical significant safety findings were considered drug related, indicating a good safety profile for this compound. Early administration of antivirals in viral respiratory infections is a pre-requisite for a successful therapeutic clinical strategy, due to the acute nature of the infection. Specifically for fusion inhibitor, the compound should be present early enough to allow the blocking of the viral entry into the host cell, the first step in the viral replication cycle.

There are some limitations associated with our studies. Though the AGM model is highly permissive for RSV replication, the viral replication is not associated with significant clinical symptoms. Moreover, whereas in rodent models the RSV infection is associated with decrease growth in absence of RSV treatment, we were not able to see a difference in the growth curves between treated and non-treated groups, probably due to the similar and intensive manipulation of the animals in these groups, manipulations that may have affected the growth curves. The objective of our work was to analyze the TMC353121 effect on viral replication, in condition of maximum TMC353121 exposure, objective achieved through administration of the compound as continuous infusion, through a central catheter. However, the use of central line infusion is limited in bronchiolitis patients to patients severely ill, in ICUs. Hence, further work will be needed to understand how the PK/PD results obtained for continuous infusion administration will translate to the clinically more acceptable modes of drug administration (i.e.: oral or bolus injection) for treatment of bronchiolitis.

Our data thus suggest that an RSV fusion inhibitor could have therapeutic effect if applied early enough after the initiation of the viral replication, similar to the currently used influenza antivirals. The replication of influenza virus in humans is shorter, with faster progression to peak VL and clinical symptoms, when compared to RSV [28,32]. Further investigations are required to understand the potential implication for the development of RSV antivirals of the longer viral shedding, prolonged time to peak clinical symptoms and specific pathology observed for RSV infection compared to influenza.

In conclusion, TMC353121 exerts a dose-dependent antiviral effect ranging from full inhibition to absence of antiviral activity, in a preclinical model highly permissive for RSV replication. The complete inhibition of viral replication was achieved for relatively low drug exposure compared with the prophylactic drug in the market (palivizumab), and in absence of compound related safety findings, positioning the RSV fusion inhibitor as a front runner in the race for RSV antivirals development.

## Supporting Information

S1 ARRIVE Checklist ARRIVE Guidelines Checklist. (PDF)

S1 Fig Correlation between viral load in BALF and throat. Relationship between viral load in lower (BALF) and upper (Throat) respiratory compartment across the two studies; r = Spearman coefficient; p< 0.0001.
(TIF)

S2 Fig Lung histopathology. Very pronounced inflammatory changes in the vehicle control groups and Px5 group, varying from minimal to marked degree, and much less pronounced changes in the Px500 group, varying from minimal to slight degree inflammation. Examples: lung histopathology for the control Vh2 and Px500 groups.
(TIF)
