# Supplementary figures and images for: Antiviral Activity of TMC353121, a Respiratory Syncytial Virus (RSV) Fusion Inhibitor, in a Non-Human Primate Model
Source: PLoS One. 2015 May 26;10(5):e0126959. doi: 10.1371/journal.pone.0126959 (PMC4444337; doi:10.1371/journal.pone.0126959)

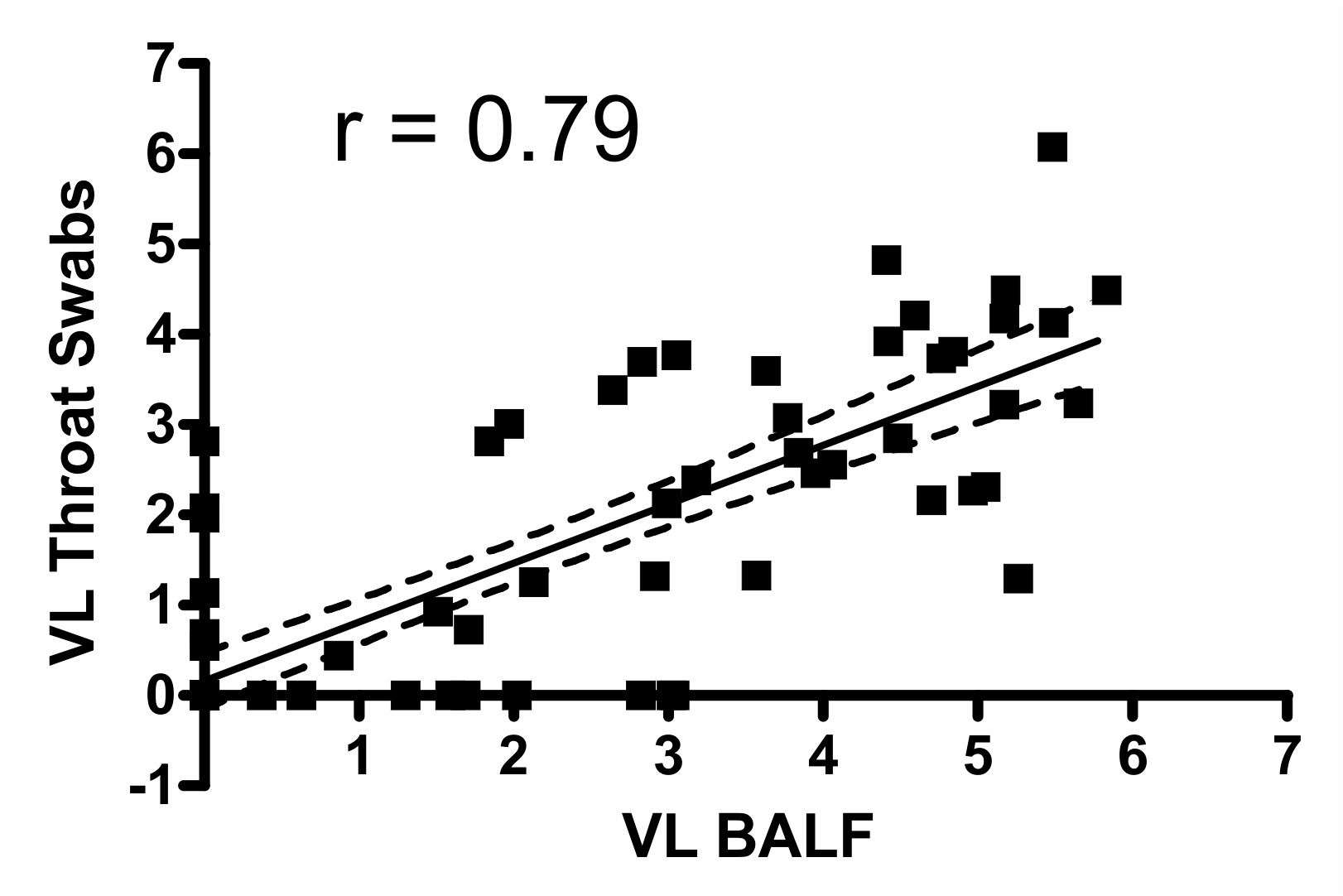

Supplement: S1 Fig — Relationship between viral load in lower (BALF) and upper (Throat) respiratory compartment across the two studies; r = Spearman coefficient; p< 0.0001. (TIF) [file pone.0126959.s002.tif]

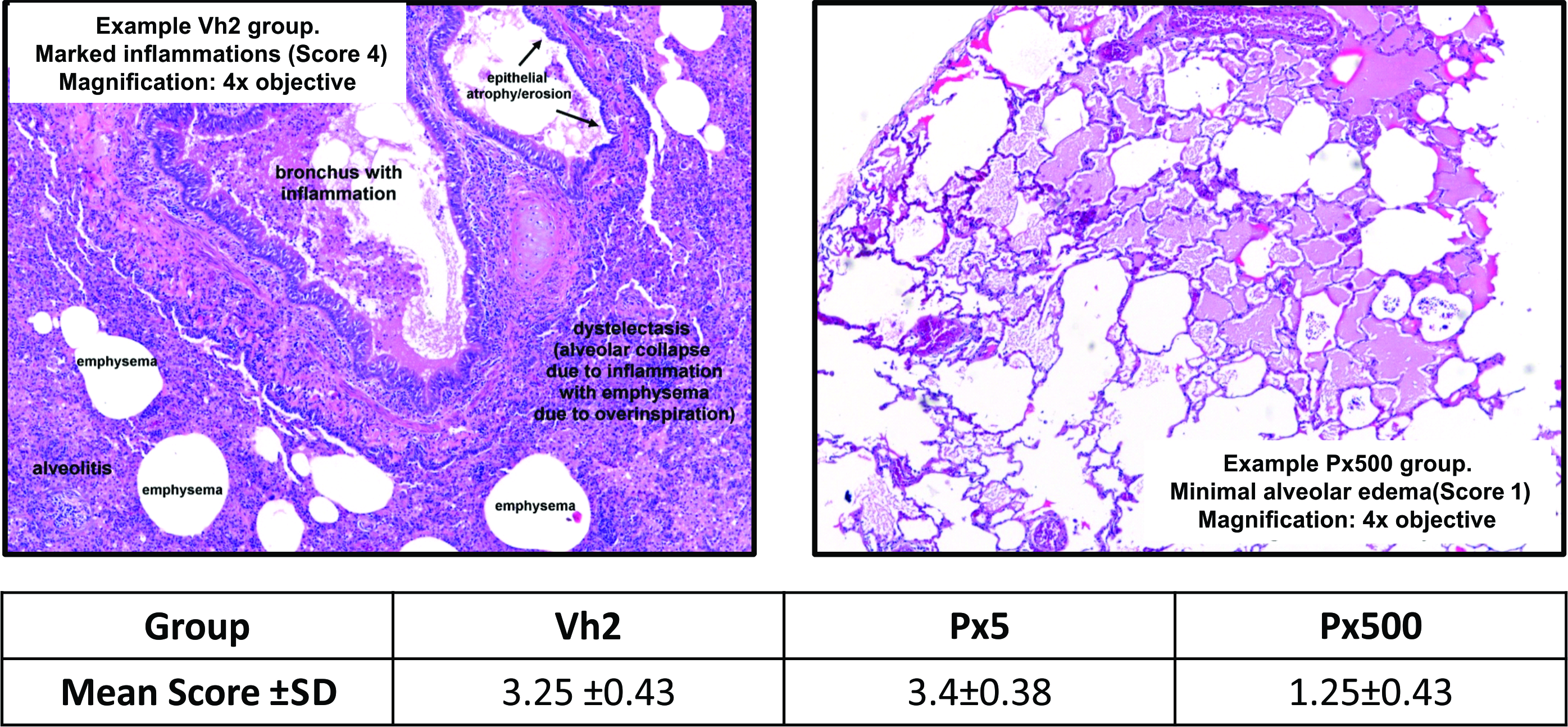

Supplement: S2 Fig — Very pronounced inflammatory changes in the vehicle control groups and Px5 group, varying from minimal to marked degree, and much less pronounced changes in the Px500 group, varying from minimal to slight degree inflammation. Examples: lung histopathology for the control Vh2 and Px500 groups. (TIF) [file pone.0126959.s003.tif]
